# Extracellular matrix and wall composition are diverse in the organogenic and non-organogenic calli of *Actinidia arguta*

**DOI:** 10.1007/s00299-020-02530-2

**Published:** 2020-03-30

**Authors:** Marzena Popielarska-Konieczna, Katarzyna Sala, Mohib Abdullah, Monika Tuleja, Ewa Kurczyńska

**Affiliations:** 1 Department of Plant Cytology and Embryology, Faculty of Biology, Institute of Botany, Jagiellonian University in Cracow, Gronostajowa 9, 30-387 Cracow, Poland; 2 Faculty of Natural Sciences, Institute of Biology, Biotechnology and Environmental Protection, University of Silesia in Katowice, Jagiellonska 28, 40-032 Katowice, Poland

**Keywords:** Callus, Kiwiberry, Immunohistochemistry, Isolated endosperm, Plant extracellular matrix, Scanning electron microscopy

## Abstract

**Key message:**

**Differences in the composition and the structural organisation of the extracellular matrix correlate with the morphogenic competence of the callus tissue that originated from the isolated endosperm of kiwifruit.**

**Abstract:**

The chemical composition and structural organisation of the extracellular matrix, including the cell wall and the layer on its surface, may correspond with the morphogenic competence of a tissue. In the presented study, this relationship was found in the callus tissue that had been differentiated from the isolated endosperm of the kiwiberry, *Actinidia arguta*. The experimental system was based on callus samples of exactly the same age that had originated from an isolated endosperm but were cultured under controlled conditions promoting either an organogenic or a non-organogenic pathway. The analyses which were performed using bright field, fluorescence and scanning electron microscopy techniques showed significant differences between the two types of calli. The organogenic tissue was compact and the outer walls of the peripheral cells were covered with granular structures. The non-organogenic tissue was composed of loosely attached cells, which were connected via a net-like structure. The extracellular matrices from both the non- and organogenic tissues were abundant in pectic homogalacturonan and extensins (LM19, LM20, JIM11, JIM12 and JIM20 epitopes), but the epitopes that are characteristic for rhamnogalacturonan I (LM5 and LM6), hemicellulose (LM25) and the arabinogalactan protein (LM2) were detected only in the non-organogenic callus. Moreover, we report the epitopes, which presence is characteristic for the *Actinidia* endosperm (LM21 and LM25, heteromannan and xyloglucan) and for the endosperm-derived cells that undergo dedifferentiation (loss of LM21 and LM25; appearance or increase in the content of LM5, LM6, LM19, JIM11, JIM12, JIM20, JIM8 and JIM16 epitopes).

## Introduction

Unorganised cell masses, which are called the callus can theoretically be produced from any living plant cell (Ikeuchi et al. 2013 and references therein). In a natural setting and under culture conditions, the induction of cell proliferation that leads to callus formation can be caused by many biotic and abiotic factors (pathogens, wounding, plant growth regulators). Because of its great plasticity the callus is valuable experimental model for basic research (Ikeuchi et al. 2017; Feher 2019; Niazan et al. 2019) as well as for industrial and bioengineering applications (Efferth 2019). The different types of calli could reveal various morphological, histological and molecular characteristics. One of the main features is their ability to form somatic embryos (in an embryogenic callus) or organs (in an organogenic callus) in the morphogenetic pathways or a lack of any obvious regeneration processes (in a non-morphogenic callus) (Ikeuchi et al. 2013 and references therein).

The statement that the composition of the cell wall is crucial for cellular differentiation is also related to the callus cells. The primary plant cell wall is a complex and dynamic structure that is predominantly composed of cellulose, hemicelluloses, pectins and structural glycoproteins (Cosgrove 2005). An increasing amount of literature data has indicated that all of these components are involved in the different developmental processes. It is postulated that pectins are involved in growth, morphogenesis, development, defence, cell adhesion and wall porosity, as well as ion or enzyme binding (reviewed in Mohnen 2008; Daher and Braybrook 2015). It was documented that the level of pectin methyl-esterification changed during somatic and microspore embryogenesis (Chapman et al. 2000; Barany et al. 2010; Sala et al. 2013; Solis et al. 2016), which indicates that they play a role in regulation of these processes. Hemicelluloses are mainly responsible for the cell wall mechanics, extensibility and cell expansion (Scheller and Ulvskov 2010); however, some may serve as storage material (Hoch 2007). Among the wall proteins, the arabinogalactan proteins (AGPs) are involved in cell differentiation, morphogenesis, plant defence and reproductive processes (for review see Showalter 2001). Some AGPs display a specific expression pattern during organ development or in an in vitro culture, where they diversify cells with different identities (Knox et al. 1991; Konieczny et al. 2007; Potocka et al. 2018). Other structural proteins group, extensins, regulate the growth and properties of the cell walls (Lamport et al. 2011). In addition, they play a role in the plant response to various abiotic and biotic stresses (Cassab 1998).

Immunohistochemical studies using the monoclonal antibodies that recognise specific epitopes might provide examples of any spatial and temporal differentiation of the wall polysaccharides or proteins in relation to growth and development (for review see Somerville et al. 2004). Moreover, such studies have also shown that various polymers are not uniformly distributed within the walls, thus indicating that the cell wall composition and structure might reflect different requirements for the elasticity or the mobility of various types of molecules in the cell wall in relation to cell differentiation and the reaction to biotic and abiotic factors. For the *Actinidia deliciosa* endosperm-derived callus, the presence of an extracellular matrix (ECM) that covers the surface of the organogenic domains might be linked to the acquisition of organogenic competence (Popielarska-Konieczna et al. 2008). The same study showed that low-methylesterified pectins and lipids are the components of the surface layer of the callus that has an organogenic capacity (Popielarska-Konieczna et al. 2008). In the maize callus, the extracellular matrix surface network (ECMS) of the embryogenic cells contained the AGP epitope, which is recognised by the JIM4 antibody; while, the non-embryogenic callus cells were devoid of this epitope (Šamaj et al. 1999, 2008). Analysis of the cell wall polysaccharide composition of the embryogenic and non-embryogenic calli that had been obtained from hypocotyl and petiole explants from *Medicago arborea* L. has revealed that the levels of total sugars, pectins, and hemicelluloses were higher in the embryogenic callus than in the non-embryogenic callus (Endress et al. 2009). In addition, during the somatic embryogenesis of *Trifolium nigrescens* Viv., it was demonstrated that the low methyl-esterified homogalacturonan (HG), which is recognised by the JIM5 antibody, and the arabinan from side chains of rhamnogalacturonan I (RG I), which is recognised by the LM6 antibody, were detected in the embryogenic sectors of the explant (Pilarska et al. 2013). Moreover, the chemical composition of the cell walls and ECM of a *Brachypodium* callus displayed spatial differences that correlated with the embryogenic character of the cells (dense cytoplasm, high nucleus/cytoplasm ratio, large nucleoli), thus indicating that the distribution of pectins, AGPs and hemicelluloses can be used as molecular markers of the embryogenic cells (Betekhtin et al. 2016).

Kiwiberry, *Actinidia arguta*, is one of the 55 species in the genus *Actinidia* (Li et al. 2009). The native distribution range of all Actinidia taxa is Asia, especially the territory of China. Only certain species such as *Actinidia arguta* can be cultivated in a moderate climate (Melo et al. 2017). In addition to its cold resistance, this genus has hairless fruits, which when ready for consumption are delicate, fragrant and rich in vitamin C, carotenoids and folic acid (Latocha 2017). Plant tissue cultures offer a wide range of new methods for improving the cultivars in the genus *Actinidia* (Wang and Gleave 2012 and references therein). One of them is to culture the isolated endosperm, which could reduce the time that is required to obtain plants with a higher ploidy.

The composition and structure of the cell wall are closely connected with the activity and developmental stage of a plant cell as well as the response to external stimuli (Seifert and Blaukopf 2010). Thus, the presented studies were conducted to verify whether the organogenic callus (OC) and non-organogenic callus (NOC) of *Actinidia arguta* differ in the chemical and structural composition of the cell walls and surface structures. The experimental systems that were used in this study enabled to compare the cell wall composition and structures that cover the OC and NOC callus surface to determine whether the structural-chemical characteristic of an apoplast could indicate different morphogenic competences. The presented data will contribute to the knowledge about the role of the chemical composition of the cell wall during the plant response to different environmental conditions, or in this case—in culture conditions.

## Materials and methods

### Plant materials and culture conditions

The fruits of the kiwiberry, *Actinidia arguta* cv. Bingo, were obtained from the plant germplasm collection at the Warsaw University of Life Sciences. The sterilisation of the fruits, the acquisition of the seeds, removal of the seed coat and embryo along with a dissection of the endosperm tissue were performed according to the protocol that was described for *Actinidia deliciosa* (Popielarska-Konieczna et al. 2008). The isolated mature endosperm tissue was used as the explants to induce either OC or NOC. The explants were transferred onto media consisting of full-strength MS (Murashige and Skoog 1962) salts and vitamins (Duchefa), 30 g/l sucrose and 6 g/l Plant Agar (Duchefa). The organogenic callus induction medium (OCIM) was supplemented with 0.5 mg/l thidiazuron (Sigma); whereas, the non-organogenic callus induction medium (NCIM) contained 2 mg/l 2,4-D and 5 mg/l kinetin. The 60-mm-diameter Petri dishes were sealed with Parafilm^®^ and kept at 25 °C in the darkness. The explants with proliferating cells were transferred onto a fresh OCIM and NCIM medium every 3 weeks and cultured under the same conditions. Observations and images were performed using a dissecting binocular microscope (Zeiss Germany, Stemi SV 11) that was equipped with a digital camera (Canon Power Shot G6). During the macroscopic observations, more than fifty samples of both types of callus (OC and NOC) were studied.

### Sample collection, histological and ultrastructural procedures

Pieces of a callus were collected after 6 weeks of the culture on the NCIM and OCIM media for the immunohistochemical, histological and scanning electron microscopy analyses. Five samples for the histological analysis and three samples for the ultrastructure and immunohistological analysis of both types of calli were studied. For the immunohistochemical analyses, the samples were fixed in a solution of 3% (w/v) paraformaldehyde (PFA), 1% (v/v) glutaraldehyde (GA) and 1% sucrose (w/v) in phosphate buffered saline (PBS), pH 7.0. Then, they were embedded in Steedman’s wax as was described in Sala et al. (2019). The sections (7-μm thick) were cut using a HYRAX M40 rotary microtome (Zeiss, Oberkochen, Germany) and collected on microscopic slides covered with poly-l-lysine (Menzel Gläser, Braunscheig, Germany). For the histological analyses, the samples were fixed in 5% (w/v) GA in 0.1-M PBS (pH 7.2), embedded in synthetic resin Technovit^®^ 7100, cut and stained with 1% toluidine blue O (TBO) according to the procedure that was described in Popielarska et al. (2006). For the scanning electron microscopy (SEM) analyses, the callus samples were prefixed in 5% (w/v) GA in 0.1-M PBS (pH 7.2) and then all of the steps of fixation and observation were performed according to the procedure that was described in Popielarska-Konieczna et al. (2008).

### Immunohistochemistry

For the immunolabelling procedure, the sections were proceeded as was described earlier (Sala et al. 2017, 2019). Briefly, the sections were de-waxed and rehydrated in an ethanol series (in PBS, v/v). The primary rat monoclonal antibodies (Plant Probes, Leeds, UK) that were used in the current study are listed in Table 1. The secondary antibody used was AlexaFluor 488 goat anti-rat (Cat. No. 112-545-003) (Jackson ImmunoResearch Laboratories, West Grove, PA, USA) and to visualise the cell wall, 0.01% (w/v) Calcofluor White (Fluorescent Brightener 28; Cat. No. F3543, Sigma-Aldrich, St. Louis, MO, USA) in PBS was applied for 10 min. The negative controls were prepared by omitting the primary antibody step. Before the immunolabelling of the hemicellulose probes (LM15 and LM21 antibodies), HG was removed from the sections via incubation in a pectate lyase (Cat. No. PRO-E0250, Prozomix Ltd., Northumberland, UK) and CAPS (*N*-cyclohexyl-3-aminopropanesulfonic acid; Cat. No. C263, Sigma-Aldrich, St. Louis, MO, USA) buffer solution (procedure according to Marcus et al. 2008). The observations and photo documentation were performed using a Nikon Eclipse Ni-U microscope equipped with a Nikon Digital DS-Fi1-U3 camera with the corresponding software (Nikon, Tokyo, Japan) at a maximum excitation wavelength of 490 nm (AlexaFluor 488) or 330 nm (Calcofluor White).

**Table 1:** List of primary rat monoclonal antibodies used in the current study

Antibody	Epitope	References
Pectins–homogalacturonan and rhamnogalacturonan I		
LM19	Unmethyl-esterified, partially methyl-esterified HG	Verhertbruggen et al. (2009a)
LM20	Methyl-esterified HG	Verhertbruggen et al. (2009a)
LM7	Partially methyl-esterified HG	Willats et al. (2001b)
LM8	Xylogalacturonan (HG)	Willats et al. (2004)
LM5	Tetrasaccharide in (1–4)-β-d-galactans (RG I side chain)	Jones et al. (1997)
LM9	Feruloylated-(1–4)-β-d-galactan	Clausen et al. (2004)
LM6	Pentasaccharide in (1–5)-α-l-arabinans (RG I side chain), may bind to AGPs	Willats et al. (1998)
LM13	Stretches of 1,5-linked arabinosyl residues	Moller et al. (2008)
LM16	Epitope associated with arabinans, may involve galactosyl residue(s) on RG backbones	Verhertbruggen et al. (2009b)
Hemicelluloses		
LM25	XLLG, XXLG and XXXG oligosaccharides of xyloglucan	Pedersen et al. (2012)
LM21	Heteromannan (mannooligosaccharides in mannan, glucomannan, galactomannan)	Marcus et al. (2010)
AGPs		
JIM4	Arabinogalactan glycoprotein	Knox et al. (1989)
JIM8	Arabinogalactan	Pennel et al. (1991)
JIM13	Arabinogalactan/Arabinogalactan protein	Knox et al. (1991)
JIM16	Arabinogalactan/Arabinogalactan protein	Knox et al. (1991)
LM2	Arabinogalactan protein	Smallwood et al. (1996)
MAC207	Arabinogalactan protein	Pennell et al. (1989)
Extensins		
LM1	Extensin/HRGP (epitope most likely includes extensin glycan components)	Smallwood et al. (1995)
JIM11	Extensin/HRGP glycoprotein	Smallwood et al. (1994)
JIM12	Extensin/HRGP glycoprotein	Smallwood et al. (1994)
JIM20	Extensin/HRGP glycoprotein	Smallwood et al. (1994)

## Results

### Tissue culture

**Fig. 1:**
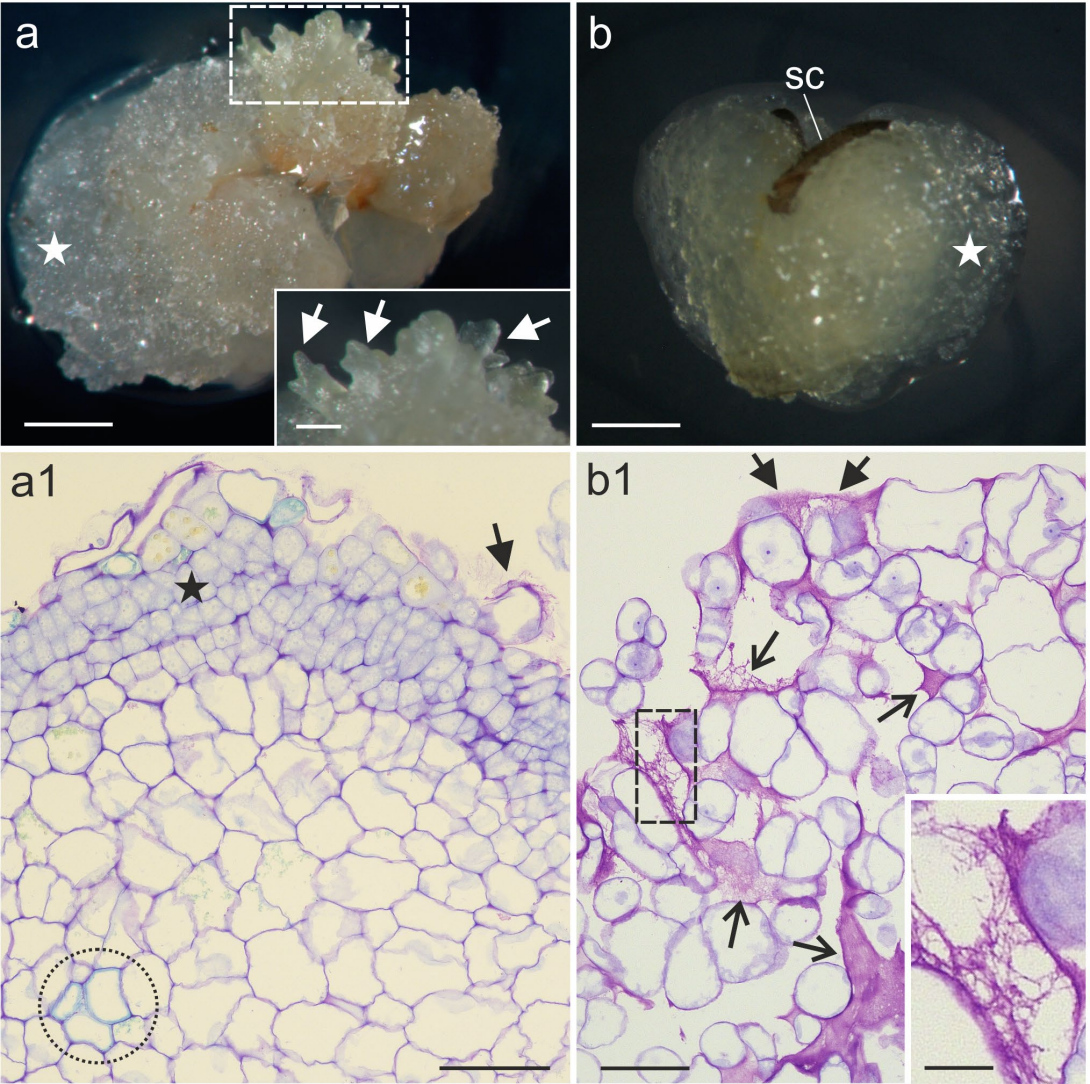
Endosperm-derived callus tissue of *A*. *arguta* after 6 weeks of the culture. **a** Callus (white star) and shoot buds (inset, arrows) on the OCIM. **a1** Histological section of the callus domain showing the compact arrangement of cells; note meristematic area (black star), tracheary elements (dotted area) and extracellularly deposited material on the surface of the cells (arrow). **b** Callus cells (white star) with remnants of the seed coat (sc) on the NCIM. **b1** Section showing loosely attached cells and fibrillar material that is visible within the large intercellular spaces (open arrow) and on the surface of the callus (arrow); magnification of the fibrillar material in inset. Scale bars: **a**, **b** = 1 mm, **a** inset = 200 µm, **a1**, **b1** = 50 µm, **b1** inset = 10 µm

**Fig. 2:**
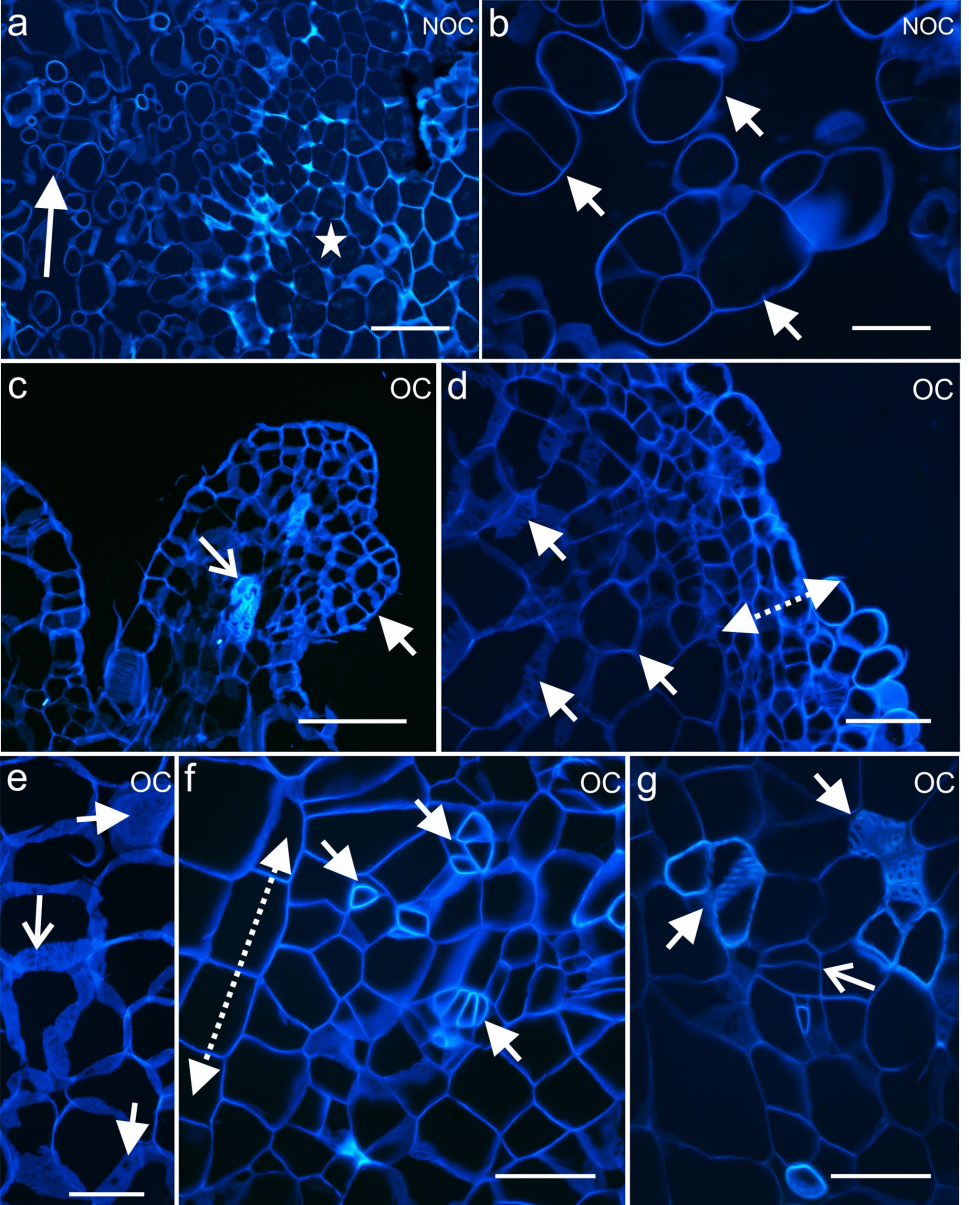
Differences in the cell organisation between the NOC—(**a**, **b**) and OC tissues (**c**–**g**), stained with Calcofluor White. **a**, **b** Loosely connected callus cells (arrows), star—endosperm cells. **c** Leaf-like structure (arrow) with tracheary elements (open arrow). **d, e** Organogenic domain on the periphery of the explant (dotted arrow) and larger callus cells in the inside of the explant with visible primary pit fields (arrows). **f** Cells of different phenotypes within an explant that show coordinated division planes (dotted arrow) and organisation (arrows). **g** Groups of tracheary elements (arrows) and cambium-like cells (arrow). Scale bar: **a**, **b** = 100 µm, **b**, **d–g** = 50 µm

After 7–10 days of the isolation of the endosperm, the first stages of callus induction were observed. The proliferation of the callus cells was observed for more than 50% and 90% of the endosperm explants on the OCIM and NCIM, respectively (data not shown). The callus that was induced on OCIM was compact and creamy in colour; whereas, the callus from the NCIM medium was white-translucent and vitreous. During the subsequent weeks of the culture, the proliferation of the callus masses continued. Protuberances of the meristematic centres and shoot buds only appeared on the OCIM medium after 5–6 weeks of the culture (Fig. 1a and inset). Histological analyses revealed a compact arrangement of the callus cells (Fig. 1a1). No morphogenic response was observed in the callus that was cultured on the NCIM medium (Fig. 1b). Sections through the callus showed loosely attached cells and intercellular spaces that were filled with fibrillar structures (Fig. 1b1 and inset). The more advanced process of the callus formation the more dispersed cells were detected (Fig. 2a). The callus cells occurred either as single or as cellular complexes (Fig. 2b). Tracheary elements were observed in the structures that had been formed from the OC (Fig. 2c). Moreover, the organogenic domains, which were localised on the periphery of an explant, differed in cell size from the cells inside the explant, which were larger and had visible primary pit fields (Fig. 2d, e). Additionally, there were a few different cell types in the OC explants (Fig. 2f, g). The cell division planes were coordinated (Fig. 2f) and the arrangement of the cells was more regular than in the NOC explants.

### SEM analysis

**Fig. 3:**
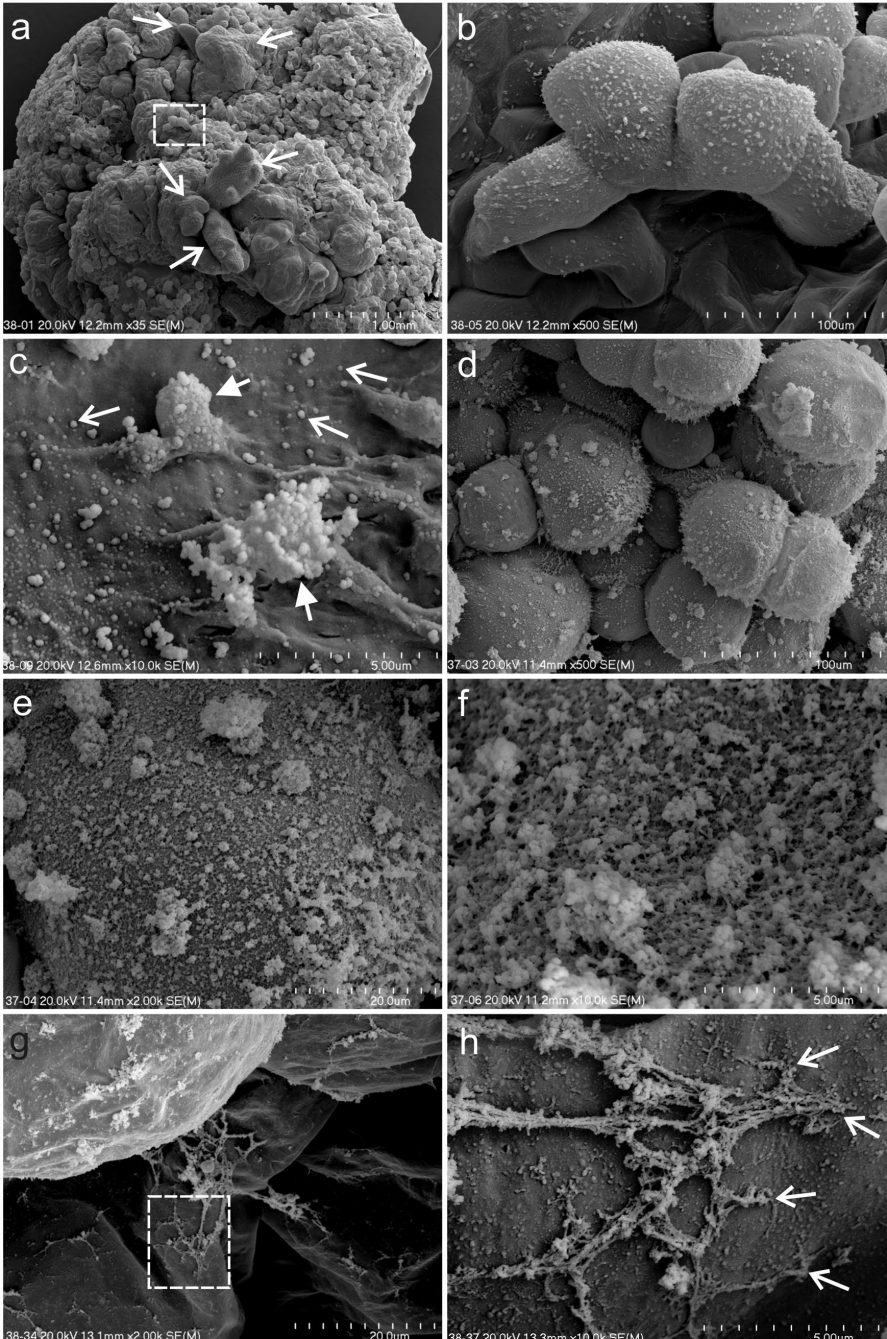
SEM micrographs of the OC of *A*. *arguta* after 6 weeks of the culture. **a** Callus domains with raised shoot buds (open arrows). **b** Magnification of the area marked by rectangle on **a** indicates the rough surface of the cell. **c** Magnification of the cell surface shows its jelly-like appearance with both single granules (open arrows) and aggregates (arrows) of granules. **d** Spherical cells with complex granular structures. **e**, **f** Magnification of the details of the cell surface from **d**. **g** Fibrillar structures on the surface and between the spherical cells. **h** Magnification of the area marked by rectangle on **g** that shows the linkages (open arrows) between the cell surface and fibrils

Observations of the OC after 6 weeks of the culture revealed shoot buds at the early developmental stages and distinct visible callus domains (Fig. 3a). A high magnification analysis revealed a mucilaginous or jelly-like appearance of the cell wall with granules: singular (approximately two nanometers in size) and aggregates that formed larger structures (Fig. 3b, c). Observations of the cells in an area with a damaged outer membranous layer (Fig. 3d) revealed a rough appearance with a more complicated structure that consisted of granules (Fig. 3e, f). The fibrous network consisted of granular components that created linkages between the cell wall within loosely attached parenchymatous cells (Fig. 3g, h).

**Fig. 4:**
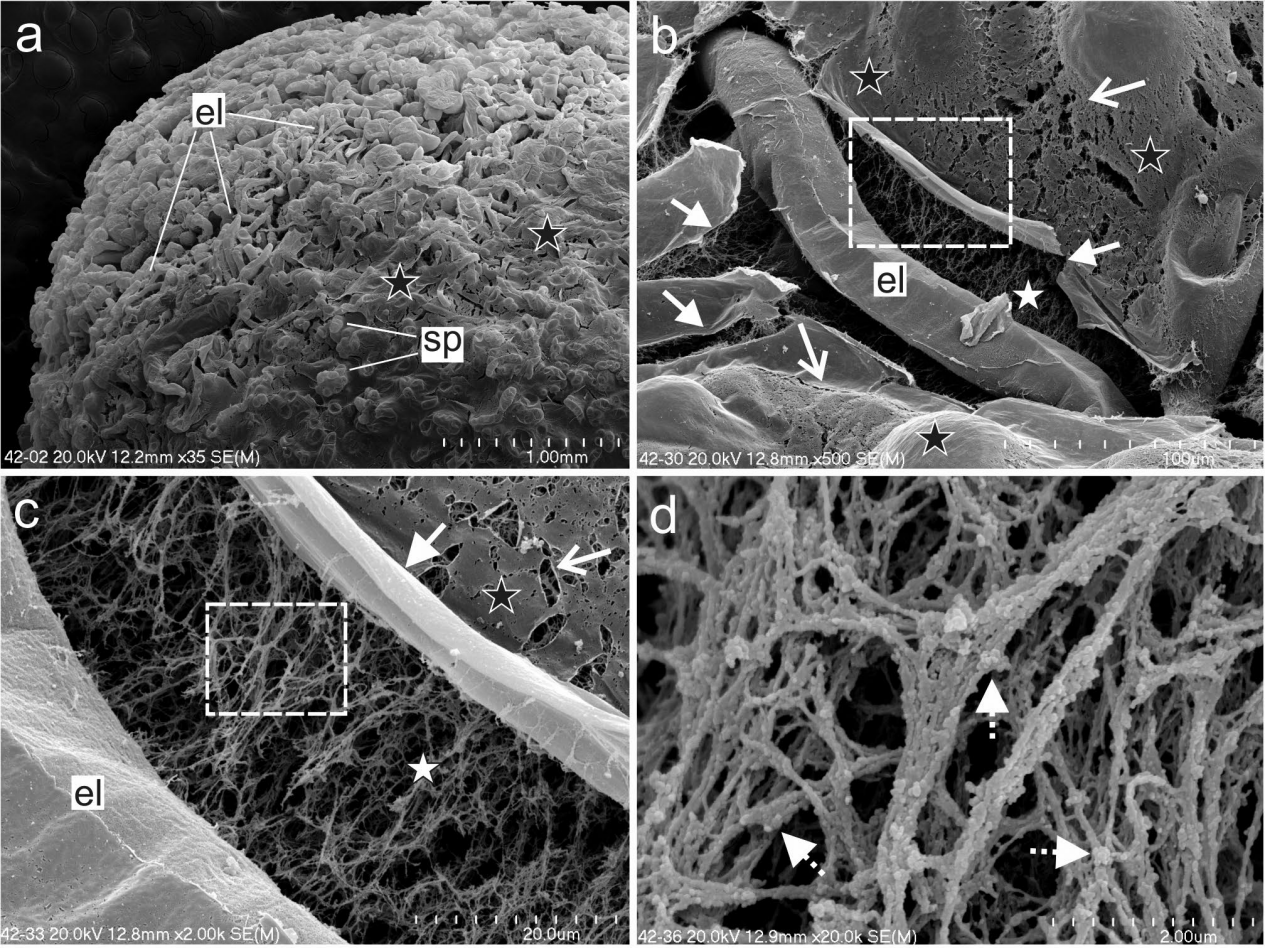
SEM micrographs of the NOC of *A*. *arguta* after 6 weeks of the culture. **a** Callus composed of parenchymatic spherical (sp) and elongated (el) cells that are covered with a membranous (black stars) layer. **b** Magnification of the membranous layer that shows the damage—small discontinuities (open arrows) and large breaks in the layer continuity (arrows). **c** Magnification of the area marked by rectangle on **b** that shows the fibrillary network under the membranous layer. **d** Magnification of the area marked by rectangle on **c** that shows granular structures that are locally associated with the fibrils (dotted arrows)

The endosperm-derived callus clumps that had been cultured on the NCIM for 6 weeks were composed of spherical and elongated cells, which were loosely attached to each other (Fig. 4a). A membranous layer, which was partially damaged, covered the surface of the callus. The damage was characterised as small discontinuities or larger breaks in the membranous layer (Fig. 4b, c), under which a dense fibrous network was observed (Fig. 4d).

### Immunohistological analysis

#### Extracellular matrix from OC and NOC cultures—differences in form and composition

**Fig. 5:**
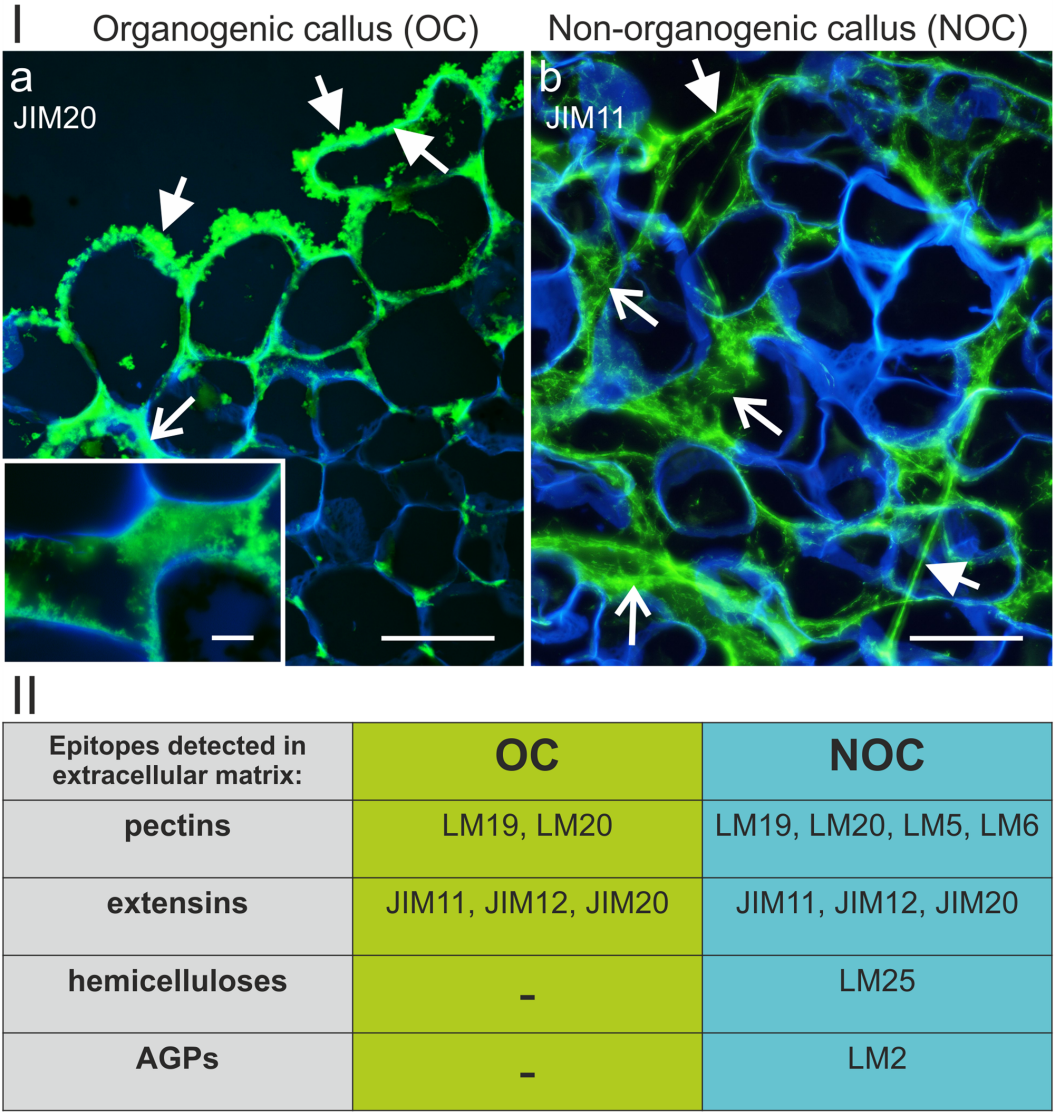
Differences between the ECM from the OC and NOC. **I** Differences in their form and localisation. **a** OC—the floccular form of the ECM that occurs at the outer walls of the peripheral cells (arrow), in the intercellular spaces (open arrow) and cytoplasmic compartments of the cells near the explant surface (arrowhead); inset: magnification of the intercellular space. **b** NOC—the ECM in a more (open arrows) or less (arrows) dispersed strands that form between the callus cells. **II** The epitopes that were detected in the ECM from the OC and NOC in which some differences in the chemical composition are visible. Scale bars: **a**, **b** = 50 µm, **a** inset = 10 µm

Analysis of the histo- and immunolabelled sections through the OC and NOC explants revealed the presence of two different extracellular matrices (Fig. 5). Among all of the analysed cell wall epitopes (listed in Table 1), nine occurred extracellularly. In the OC, the ECM had a floccular form and was deposited on the surface of the peripheral callus cells (Fig. 5a). In addition, it was detected in the intercellular spaces between the cells near the explant periphery (Fig. 5a). In the NOC, the ECM had a strand (more or less dispersed) form and seemed to connect the callus cells not only at the periphery but also within the explant (Fig. 5b). The chemical composition (in terms of the occurrence of the analysed epitopes) also differed to some degree. Un/low methyl-esterified and methyl-esterified HG (epitopes LM19 and LM20, respectively) and extensins (epitopes JI M11, JIM12 and JIM20) occurred in both the OC and NOC ECM. However, other pectic epitopes such as LM5 and LM6 (galactan and arabinan from the side chains of RG I), xyloglucan epitope LM25 and AGPs epitope LM2 were detected only in the NOC (Fig. 5, section II).

**Fig. 6:**
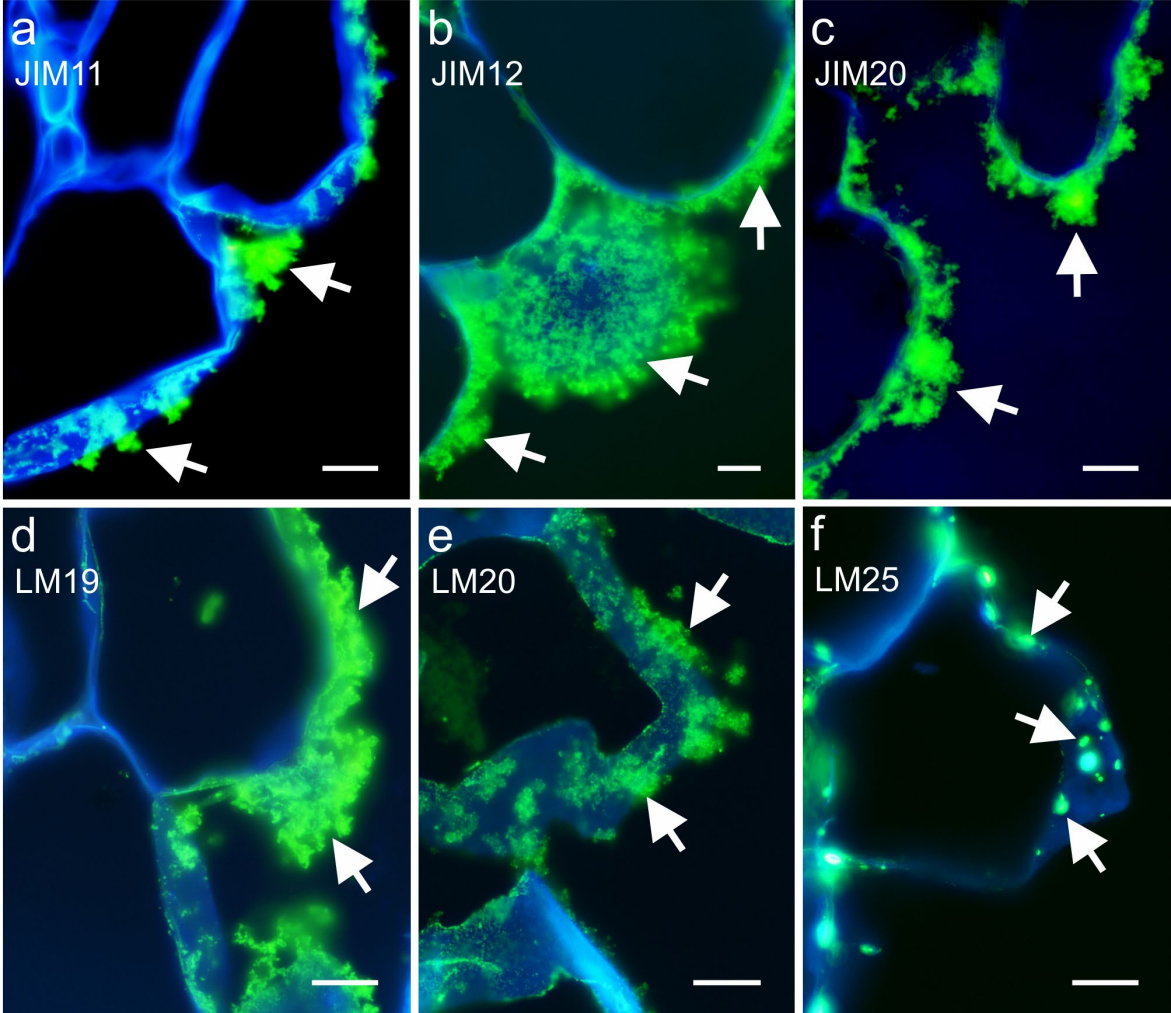
OC—abundant occurrence of extensins (**a**–**c**) and the pectic (**d** and **e**) epitopes in the floccular ECM (arrows). **f** The xyloglucan epitope, which was detected only in the patches within the outer cell wall (arrows). Scale bars: **a**–**h** = 10 µm

**Fig. 7:**
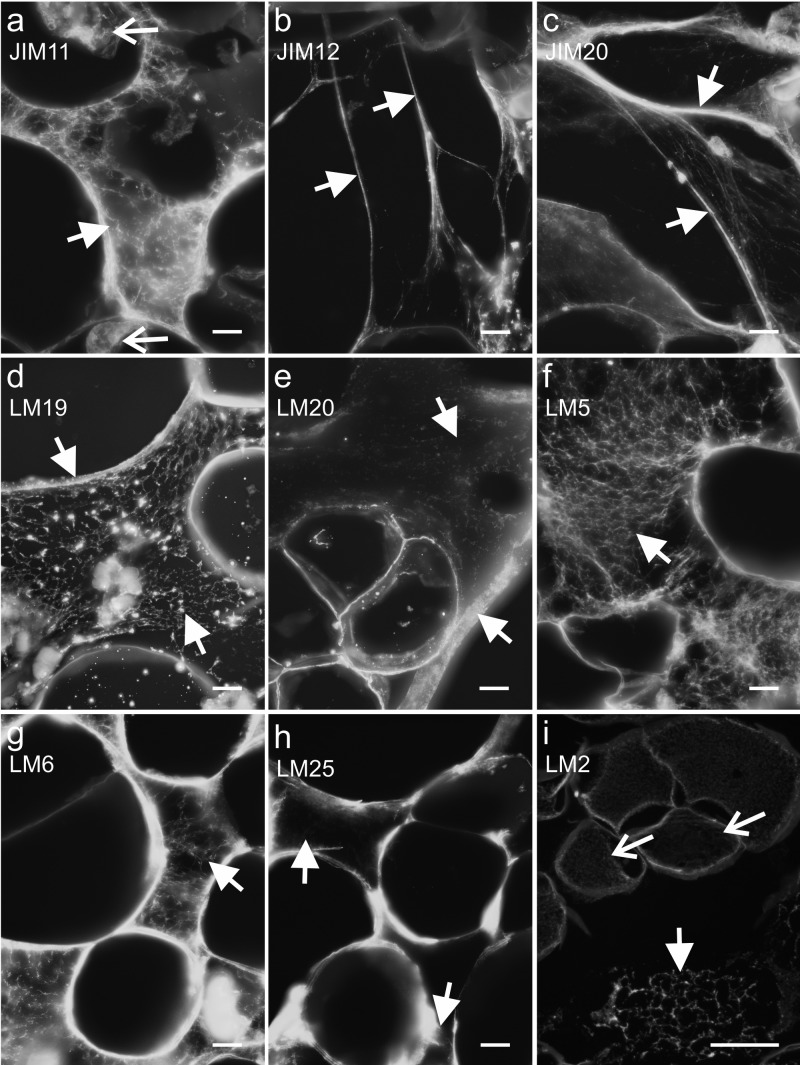
NOC—presence of extensins (**a**–**c**), pectic (**d**–**g**), hemicellulose (xyloglucan, **h**) and AGPs (**i**) epitopes in the ECM (arrows). Open arrows: **a**, **i** an epitope that was detected in the cytoplasmic compartments. Scale bars: **a**–**h** = 10 µm, **i** = 50 µm

The floccular organisation of the OC ECM and the detection of epitopes mentioned above are presented in Fig. 6 (Fig. 6a–e). It is worth mentioning that the occurrence of the LM19 epitope was more apparent than that of the LM20 epitope (Fig. 6d, e). Moreover, xyloglucan epitope LM25 was localised within the walls of the surface callus cells in a patch-like manner, but it did not appear to be a part of the ECM (Fig. 6f). The occurrence of cell wall epitopes in the NOC ECM is presented in Fig. 7.

### Endosperm and differentiation

**Fig. 8:**
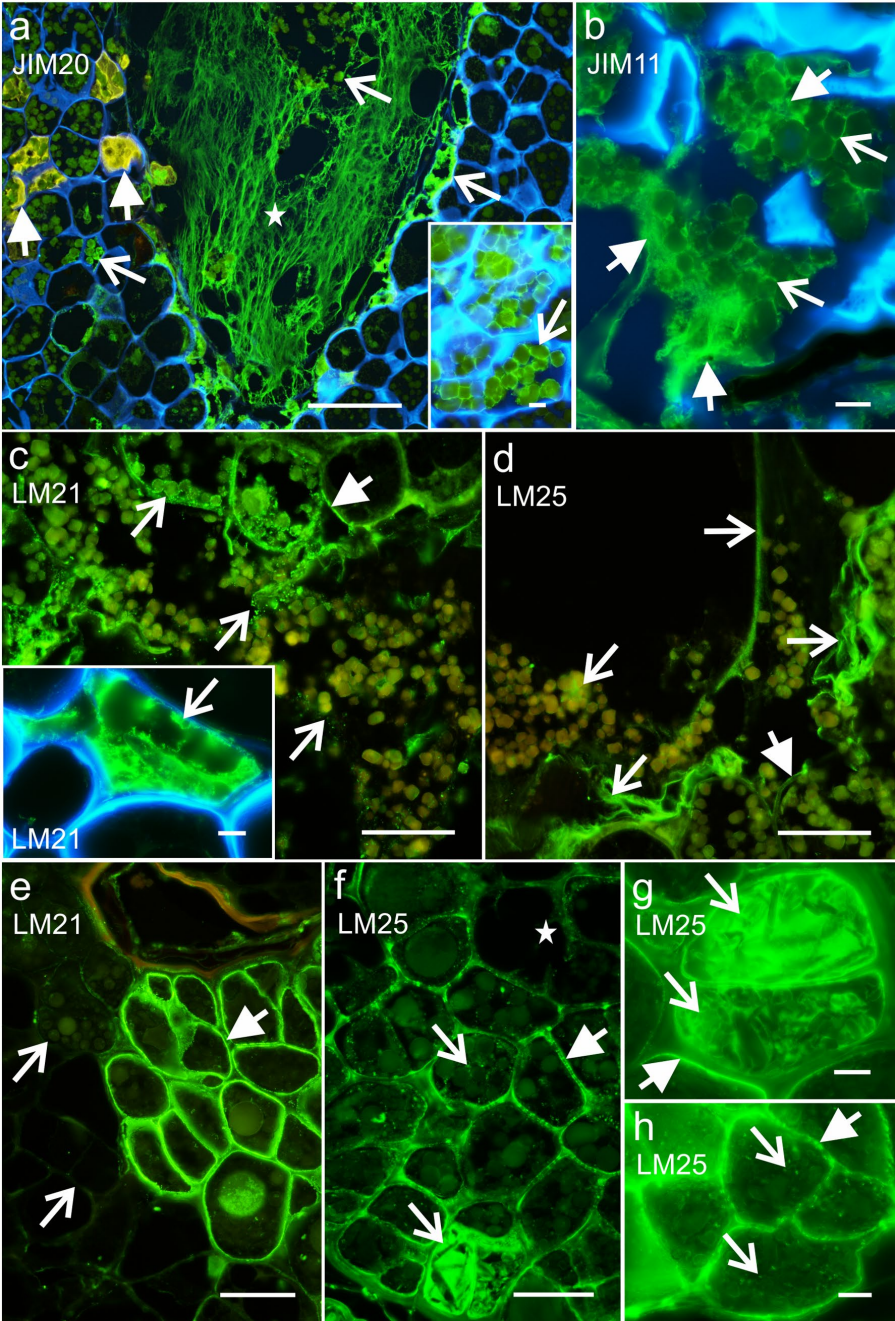
OC—the occurrence of the cell wall epitopes in the endosperm and its derivatives. **a** and inset An extensin epitope that was detected in the strands of the ECM (star), in the cytoplasmic compartments and is associated with the storage proteins (open arrows); arrows—autofluorescence of the lipid substances. **b** An extensin epitope that was detected in the ECM (arrows) and is associated with the storage proteins (open arrows). **c** A heteromannan epitope that was present in the cell walls (arrows) and is associated with the storage proteins and the ECM (open arrows); inset: an epitope that was detected in the cytoplasmic compartments. **d** A xyloglucan epitope that was detected extracellularly (open arrows) and in the cell walls (arrow). **e** The abundant occurrence of the heteromannan epitope in the endosperm cell walls (arrow) and weak fluorescence signal in the walls of its derivatives and callus cells (open arrows). **f**–**h** A xyloglucan epitope that was present in the cell walls (arrow) and cytoplasmic compartments (open arrows) of the endosperm; star—weak/no fluorescence signal in the callus cells. Scale bars: **a** = 100 µm, **c**, **d**, **e**, **f** = 50 µm, **a** inset, **b**, **c** inset, **g**, **h** = 10 µm

Two morphological domains could be distinguished in the OC—organogenic, which was localised on the explant periphery and a callus with a different organisation than in the NOC (Figs. 1, 2). Moreover, undifferentiated cells of the endosperm and cells that had begun to divide and differentiate (hereafter called “derivatives”) were also detected. Regardless of the OC ECM that covered the surface of the explant, another type of ECM was observed in the vicinity of the endosperm cells and their derivatives (Fig. 8), in which epitopes belonging to the extensins (JIM11, JIM12 and JIM20) and hemicelluloses (heteromannan: LM21, xyloglucan: LM25) were detected. The extensin epitopes were detected not only extracellularly, but also in the cytoplasmic compartments where they were associated with storage proteins (Figs. 8a, b, JIM12 not shown). Epitopes LM21 and LM25 had a similar localisation and they occurred abundantly in the walls of the endosperm cells (Fig. 8c–h). In the endosperm derivatives, the LM21 and LM25 epitopes were absent (or present in a low amount, Fig. 8e, f). The presence of the LM25 epitope in the endosperm cytoplasmic compartments was observed either as a strong (Fig. 8f, g) or punctate (Fig. 8f, h) fluorescence signal.

**Fig. 9:**
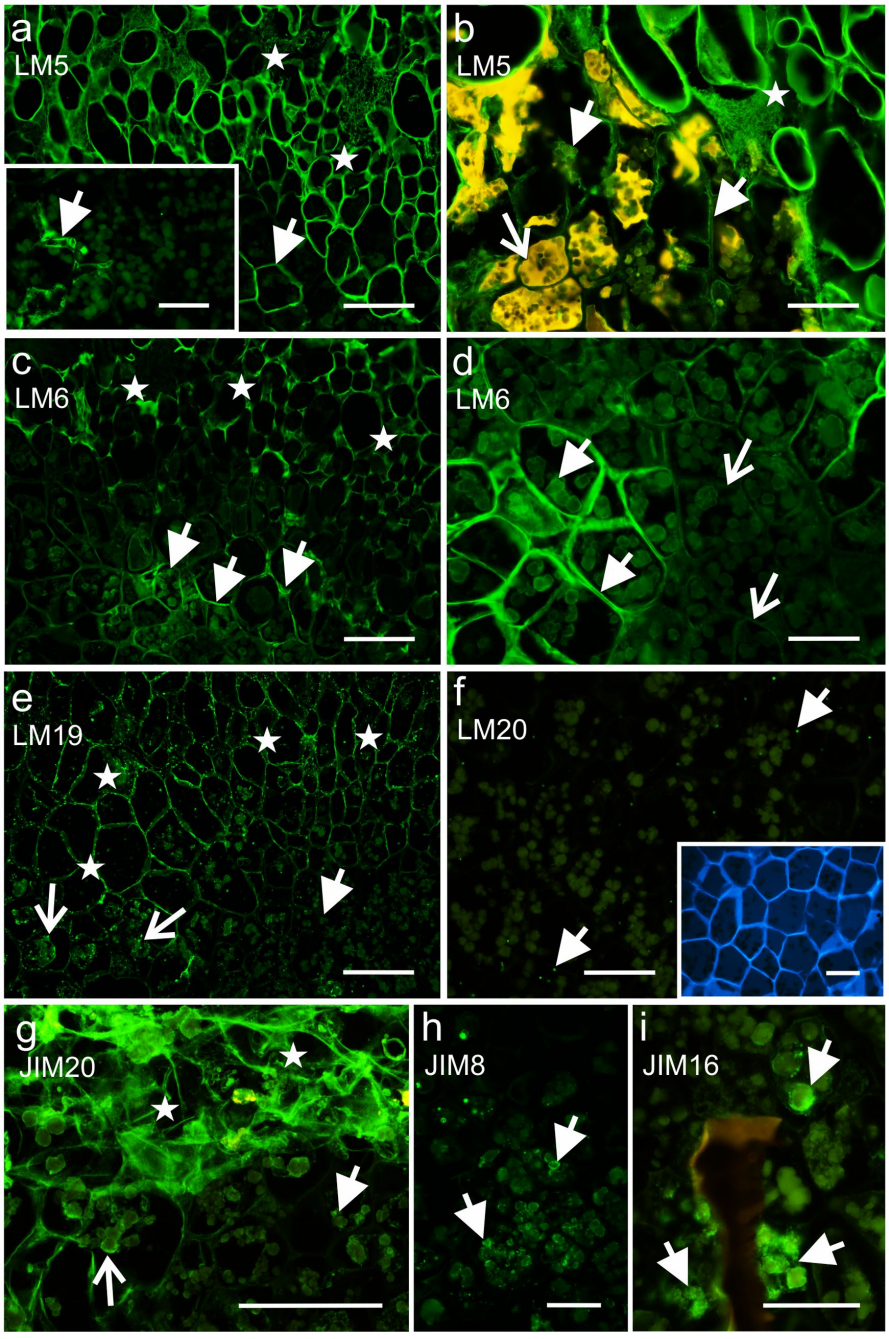
NOC—detection of the wall epitopes in endosperm and callus cells. **a** and inset, **b** The abundant occurrence of the galactan epitope in the walls of some endosperm cells, cytoplasmic compartments and intercellular spaces (arrows), the strong fluorescence signal in the callus cells and ECM (stars), open arrow—autofluorescence of the lipid substances. **c**, **d** The presence of the arabinan epitope in the walls, cytoplasmic compartments and intercellular spaces of some endosperm cells (arrows), open arrows—weak/no fluorescence signal in the endosperm cells, stars—a strong fluorescence signal in the callus cells and ECM. **e** A moderate presence of the HG epitope in the endosperm cells (arrow), the abundant occurrence of its derivatives in the cytoplasmic compartments (open arrows), in the cell walls and ECM (stars). **f** Absence of the HG epitope in the endosperm except for a punctate fluorescence signal (arrows), inset: calcofluor. **g** An extensin epitope that was detected in the storage proteins of the endosperm cells (arrow) and its derivatives (open arrow), stars—the abundant occurrence in the callus cells and ECM. **h**, **i** The AGPs epitopes that were detected in some of the storage proteins in some endosperm cells (arrows). Scale bars: **a**, **c**, **e**, **g** = 100 µm, **a** inset, **b**, **d**, **f**, **f** inset, **h**, **i** = 50 µm

In the endosperm of NOC explants, the epitopes that are recognised by the LM5 and LM6 antibodies had a varied localisation (Fig. 9a–d). The LM5 and LM6 epitopes were only observed in a part of the endosperm cell walls, the cytoplasmic compartments or intercellular spaces (Fig. 9a–d) and the more advanced the stage of cell differentiation, the more pronounced this occurrence was. However, the un/low methyl-esterified HG (recognised by LM19 antibody) was detected in a moderate amount in the endosperm cells and the amount of epitope increased in its derivatives (Fig. 9e). There was no methyl-esterified HG (LM20 antibody) in the endosperm cells (Fig. 9f). Like LM19, the extensin epitopes were observed in the endosperm cells at a moderate level, and were associated with the storage proteins (Fig. 9g, JIM11 and JIM20 not shown), but they became more abundant in the callus cells (Fig. 9g). Moreover, two AGPs epitopes, that are recognised by the JIM8 and JIM16 antibodies, were detected in the cytoplasmic compartments of some of the endosperm cells (associated with storage proteins, Fig. 9h, i).

## Discussion

### General ultrastructure and histology of a callus with a diverse morphogenic capacity

The ability of isolated endosperm tissue to proliferate and regenerate has been proved for a wide range of species (Hoshino et al. 2011; Wang et al. 2016; Winarto et al. 2018) including the genus *Actinidia*, like *A. deliciosa* (Goralski et al. 2005) or *A. kolomikta* (Asakura and Hoshino 2017). The experimental system that had previously been established for obtaining the endosperm-derived callus in *A. deliciosa* (Goralski et al. 2005) was also found to be appropriate for *A. arguta* (not published). The culture conditions that were used led to the development of a callus with the ability to regenerate shoot buds or only to proliferate. A significant advantage of the isolated endosperm in *A. arguta* is the simultaneous induction of the explants and the growth of the OC or NOC callus in contrast to *A. deliciosa.* Thus, *A. arguta* cultures offer a unique possibility to conduct research on tissues that are differentiated from the same type of primary explant (isolated endosperm) and are at the same age, but have different abilities for the morphogenic processes under diverse treatments.

In presented study, the NOC was composed of mass of unorganised, loosely packed cells with no sign of any cyto-histological differentiation. By contrast, histological differentiation was detected in the the OC. Many tracheary nodules were present in the explant, there were numerous cell divisions of the explant cells, and finally, the development of shoot-like structures was observed. The differentiation of the tracheary elements is often observed within the callus, especially in domains that have the capacity to form organs (Govil et al. 2017). The non-organogenic callus had a friable appearance with loosely attached cells that differed in size and shape. Histological observations of the extended ECM, which was visible as a net-like component that filled the intercellular spaces and the layer covering the surface of the callus, have been observed in the callus of other species such as *Zea mays* (Šamaj et al. 1999), *A. deliciosa* (Popielarska et al. 2006) or *Rumex sp.* (Ślesak et al. 2014). The net-like structure that was observed in the intercellular spaces of the non-organogenic callus corresponded to the dense fibrillar structures that were clearly visible at the ultrastructural level. These specific “constructions” among the spaced cells in the callus with no morpho- or organogenic capacity could be involved in creating a system that connects individual cells (Iwai et al. 2001). Similar results concerning the histological diversity among callus cells was described for other plants such as *Piper nigrum* (Sujatha et al. 2003), *Populus euphratica* (Ferreira et al. 2009), *Cichorium intybus* (Dakshayini et al. 2016), *Corylus avellana* (Silvestri et al. 2016), and many others (for review see Fehér 2019). Thus, the morpho-histological analysis of the callus presented in this paper is in accordance with the literature data.

The surface of the cells in the OC was distinguished by a mucilaginous or jelly-like layer with numerous globular particles, which were able to form aggregates, or that were a component of fibrillar strands. The hydrated polymers including polysaccharides had a specific appearance during the SEM analyses. The decrease in the number of water molecules during the fixation and critical point drying (CPD) procedure could affect the structural transformation of the chemical components. This phenomenon relates to hydrated pectins, which are a main component of the primary cell wall (Muscariello et al. 2005; Pathan et al. 2008; Bidhendi and Geitmann 2016; Broxterman and Schols 2018). Additionally, it is possible that proteins such as extensins, which may be covalently linked with the wall polysaccharides (Fruleux et al. 2019 and references therein), affect the organisation of the pectins and finally have an influence on the appearance after CPD treatment. A different appearance of the external layer in the SEM analyses, which was dependent on the SEM procedure, was described earlier (Popielarska-Konieczna et al. 2010). Damage to the membranous layer that covers the callus cells in *A*. *deliciosa* was observed especially after using the CPD. Moreover, during the morphogenic processes in the kiwifruit callus, it was observed that the ECM disappeared along with the cutin formation (Popielarska-Konieczna et al. 2011).

### Chemical composition of ECM and cell wall as a marker for different cell fates

The cell wall constituents can be markers of the developmental program that is implemented by cells. The modification, re-organisation, synthesis and deposition of specific wall components are processes that are closely correlated with changes in the cell fate (Fry 1995; Somerville et al. 2004; Kurczynska et al. 2012). Firstly, the obtained results indicate that the chemical composition and structure of the ECM in the OC and NOC are different. The question of whether the chemical composition of the ECM is a general feature of the regenerative capacity of some callus cells or whether it is a characteristic of the cells that are committed to a specific developmental program, organogenic or non-organogenic, arises. An analysis of the ECM composition in a wheat callus suggested the first possibility (Konieczny et al. 2007). The ECM had similar features during the shoot and embryo development (Konieczny et al. 2005, 2007). On the other hand, studies on the *Papaver somniferum* L. showed that the character of the surfaces that cover the callus cells depends on the specific stages of the regeneration and morphogenic program (Ovećka and Bobák 1999). The pectic epitopes that are recognised by the LM19 and LM20 antibodies were present in the ECM of each callus type. Although both LM19 and LM20 bind to the HG domains, only LM19 can recognise unmethyl-esterified HG (Verhertbruggen et al. 2009a). It has been postulated that the residues of galacturonic acid that are present in the chains of low or unmethyl-esterified HG are cross-linked with calcium cations, thus forming a “pectin gel”, which, in turn, may contribute to wall stiffening (Willats et al. 2001a; Jiang et al. 2005; Caffall and Mohnen 2009; Hongo et al. 2012). The presence of the above-mentioned pectic epitopes in the ECM has been observed not only on the surface of the somatic embryos and embryogenic callus but also on the OC in different plant species (Konieczny et al. 2007; Popielarska-Konieczna et al. 2008; Betekhtin et al. 2016). Chapman et al. (2000) stated that the non-esterified pectins that are present in the extracellular matrices may be responsible for maintaining embryonic cell adhesion and that the occurrence of the layer that covers the cell itself could spatially limit cell division. It is worth mentioning that the other pectic epitopes that were analysed during the presented studies such as those that are recognised by the LM7, LM8, LM13 and LM16 antibodies, were absent, regardless of the morphogenic capacity of the callus.

Extensins are hydroxyproline-rich glycoproteins of a basic charge that cross-linking into network and strengthen the structure of the cell wall (Lamport et al. 2011). It was shown that extensins may play a role in the acquisition of resistance to pathogens by reinforcing the wall structure (Ribeiro et al. 2006). Moreover, extensins self organise into a network in in vitro conditions and may interact with other wall components that have an opposite acidic charge (Cannon et al. 2008; Lamport et al. 2011). Thus, these proteins can affect the pectin properties and the degree of cell wall hydration (MacDougall et al. 2001; Pereira et al. 2011). In our study, three extensin epitopes that are recognised by the JIM11, JIM12 and JIM20 antibodies were detected in the extracellular matrices from the OC and NOC as well. Based on the data given above, a protective or cell-associating function can be proposed. Additionally, such an extracellular localisation of the extensins is not unique—extensins were detected on the surface of Brachypodium callus cells (Betekhtin et al. 2016) and in an Arabidopsis graft union (Sala et al. 2019).

However, similarities in the chemical composition between the apoplast components of the OC and NOC (in terms of the analysed epitopes) end at this point. In contrast to the OC, epitopes such as LM5 and LM6 (both occurring within the pectin, RG-I, galactan and arabinan side chains, respectively), LM25 (xyloglucan) and LM2 (AGP epitope) were found in the NOC ECM. The RG-I side chains such as arabinan and galactan are modified by different biotic and abiotic factors (Baldwin et al. 2014; Muschitz et al. 2015; Tenhaken 2015), but were not analysed in the callus cells that were undergoing different developmental pathways. Although the function of the arabinan and galactan side chains of pectin has not yet been fully determined (Ha et al. 2005), it has been postulated that a high arabinan content is responsible for the rehydration of the cell wall (Tenhaken, 2015) and that this probably plays the role of a pectic plasticiser to keep the cell wall flexible (Moore et al. 2013; Tenhaken 2015). The galactan side chains of RG-I are postulated as being the component that maintains cell stiffness (McCartney et al. 2001). However, the molecular interaction between the components that occurs in the ECM of the OC and NOC and their physical properties must be investigated in future studies.

Moreover, when the differentiation of the endosperm cells began, some of the epitopes that are characteristic for endosperm cells such as LM25 (epitopes within the xyloglucan chain) and LM21 (epitopes from heteromannan: mannan, glucomannan and galactomannan) were absent from the endosperm derivatives and callus cells, while some did appear (LM5, LM6, LM19, JIM11, JIM12, JIM20, JIM8 and JIM16), which clearly indicates the occurrence of cells with an altered cell wall composition during the culture. It was shown that xyloglucan occurs as storage material, the so-called amyloid, in the cotyledon cell walls of many plant species (Buckeridge et al. 1992). Other hemicelluloses, mannan and galactomannan, may occur as a reserve material in the walls or vacuoles of the endosperm from various plants (Matheson 1990; Buckeridge et al. 2000). Thus, the loss of LM25 and LM21 epitopes may be an expression of cell differentiation and may indicate that the derivatives begin to realise a different developmental program, which means further cells divisions that lead to, e.g. callus cells.

The signals within the cytoplasmic compartments that are associated with the storage proteins are of particular interest. There is a fluorescence signal at the protein surface and in the threads that are attached to the endosperm and callus cells. Are extensins part of these proteins and are they activated to form a network? In addition, could the appearance of AGPs, which are considered to be signalling proteins, be early markers of cell differentiation? To answer these questions, it will be necessary to analyse the proteome during the changes of the endosperm cell fate using a biochemical approach.

Such a detailed analysis of the spatio-temporal chemical composition of the ECM will contribute to the knowledge about the surface layer that covers a callus that has a different morphogenic potential (Šamaj et al. 2008; Endress et al. 2009; Betekhtin et al. 2019). These results are presented for the first time (at least to the best of our knowledge) for the *A. arguta* callus. Moreover, the obtained results provide new data for the in vitro cultures of a species that is important from an agronomic point of view.

## Author contribution statement

MPK devised the research. KS, MA and MT performed the experiments. KS, EK and MPK interpreted the results. KS, MA and MPK designed the figures. MPK, KS and EK wrote the manuscript. All of the authors read and approved the manuscript.

## Abbreviations

AGPs: Arabinogalactan proteins
CAPS: *N*-Cyclohexyl-3-aminopropanesulfonic acid
ECM: Extracellular matrix
GA: Glutaraldehyde
HG: Homogalacturonan
HRGP: Hydroxyproline-rich proteins
MS: Murashige and Skoog salts
NCIM: Non-organogenic callus induction medium
NOC: Non-organogenic callus
OC: Organogenic callus
OCIM: Organogenic callus induction medium
PBS: Phosphate-buffered saline
RG-I: Rhamnogalacturonan I
SEM: Scanning electron microscopy
TBO: Toluidine blue O
